# Interferon Regulatory Factor 8-Deficiency Determines Massive Neutrophil Recruitment but T Cell Defect in Fast Growing Granulomas during Tuberculosis

**DOI:** 10.1371/journal.pone.0062751

**Published:** 2013-05-24

**Authors:** Stefano Rocca, Giovanna Schiavoni, Michela Sali, Antonio Giovanni Anfossi, Laura Abalsamo, Ivana Palucci, Fabrizio Mattei, Massimo Sanchez, Anna Giagu, Elisabetta Antuofermo, Giovanni Fadda, Filippo Belardelli, Giovanni Delogu, Lucia Gabriele

**Affiliations:** 1 Department of Veterinary Medicine, University of Sassari, Sassari, Italy; 2 Department of Hematology, Oncology, and Molecular Medicine, Istituto Superiore di Sanità, Rome, Italy; 3 Institute of Microbiology, Catholic University of the Sacred Heart, Rome, Italy; Institut de Pharmacologie et de Biologie Structurale, France

## Abstract

Following *Mycobacterium tuberculosis* (*Mtb*) infection, immune cell recruitment in lungs is pivotal in establishing protective immunity through granuloma formation and neogenesis of lymphoid structures (LS). Interferon regulatory factor-8 (IRF-8) plays an important role in host defense against *Mtb*, although the mechanisms driving anti-mycobacterial immunity remain unclear. In this study, IRF-8 deficient mice (IRF-8^−/−^) were aerogenously infected with a low-dose *Mtb* Erdman virulent strain and the course of infection was compared with that induced in wild-type (WT-B6) counterparts. Tuberculosis (TB) progression was examined in both groups using pathological, microbiological and immunological parameters. Following *Mtb* exposure, the bacterial load in lungs and spleens progressed comparably in the two groups for two weeks, after which IRF-8^−/−^ mice developed a fatal acute TB whereas in WT-B6 the disease reached a chronic stage. In lungs of IRF-8^−/−^, uncontrolled growth of pulmonary granulomas and impaired development of LS were observed, associated with unbalanced homeostatic chemokines, progressive loss of infiltrating T lymphocytes and massive prevalence of neutrophils at late infection stages. Our data define IRF-8 as an essential factor for the maintenance of proper immune cell recruitment in granulomas and LS required to restrain *Mtb* infection. Moreover, IRF-8^−/−^ mice, relying on a common human and mouse genetic mutation linked to susceptibility/severity of mycobacterial diseases, represent a valuable model of acute TB for comparative studies with chronically-infected congenic WT-B6 for dissecting protective and pathological immune reactions.

## Introduction

Susceptibility to tuberculosis (TB) is known to be influenced by both genetic variation and nature of immune response [1]. Increased pulmonary lymphocyte infiltration following *Mycobacterium tuberculosis* (*Mtb*) infection is a crucial event leading to the generation of anti-mycobacterial immunity. Recent observations indicate that *Mtb* induces neogenesis of lymphoid structures (LS) in infected lungs [2]. LS are defined as ectopic lymphoid-cell accumulations that arise in non lymphoid tissues under inflammatory conditions and tightly resemble an inducible form of bronchus-associated lymphoid tissue, where local immune responses occur [3]. The formation and the maintenance of LS in chronic inflammatory conditions is critically dependent on lymphoid chemokines such as CCL19 and CXCL12 [4]. Although studies in LTα-deficient mice have suggested a role for LS in the development of acquired resistance to *Mtb* infection, their function during TB remains unclear [5].

Both innate and adaptive responses concur to generate anti-mycobacterial immunity preventing TB and are dependent on the development of granulomas [6], [7]. Granuloma formation occurs upon pulmonary inflammation due to dynamic interaction of bacilli with macrophages and the succeeding/following recruitment of monocytes, neutrophils, dendritic cells (DC), T and B cells [8], [9]. Herein, interactions between innate and adaptive immune components occur at the interface between the macrophage-rich inner layer and the surrounding outer layer enriched predominantly with CD4^+^ T cells or in follicle-like structures [10], [11]. Thus, the composition of immune infiltrates in granuloma lesions may be associated with either replication of mycobacteria during active TB, or, on the contrary, with growth control in the case of latent TB [12]. Therefore, understanding the specific contribution of lung immune infiltrates to the generation of anti-mycobacterial immunity will provide a better knowledge of TB immunobiology facilitating the design of new therapeutic strategies.

Interferon regulatory factor-8 (IRF-8) is a lineage-specific transcription factor of myeloid cells playing a critical role in both innate and acquired immunity [13], [14], [15]. It is expressed at high levels in some DC subsets and to a lesser extent in other immune cells, including granulocytes, thus controlling their functional development [16], [17]. Mice lacking IRF-8 (IRF-8^−/−^) display multiple alterations of the myeloid compartment, including defective differentiation of plasmacytoid DC and CD8α^+^ DC and systemic expansion of granulocytes [16], [18], [19]. Consequently, IRF-8^−/−^ mice are highly susceptible to most infectious agents, whose clearance depends on the induction of a competent Th1 immune response [20], [21]. BXH-2 mice, which bear a defective IRF-8R394C allele, are extremely susceptible to *M. bovis* Bacillus Calmette-Guérin (BCG) and *Mtb* infections [22]. Specifically, BXH-2 mice cannot contain aerosol *Mtb* infection due to inability of macrophages to limit bacterial replication [23]. Recently, IRF-8 deficiency has been described as the cause of a new syndrome in humans affecting monocytes/DC cell content and BCG susceptibility [24].

In this study we investigated the immune events controlled by IRF-8 at pulmonary level in host defense against TB. We show that fatal acute TB occurring in IRF-8^−/−^ mice following aerosol *Mtb* infection is associated to uncontrolled growth of pulmonary granulomas and impaired development of LS. These events are correlated with a progressive decrease of infiltrating T cells and a persistence of neutrophils associated to unbalanced expression of lymphoid chemokines at late stages of infection. Our results reveal a new important function of IRF8 as a regulator of pulmonary immune dynamics required to elicit a protective anti-mycobacterial immune response.

## Materials and Methods

### Ethics statement

Mice were maintained and treated in accordance with Legislative Decree 116/92 guidelines based on European Directive 86/609/EEC. Animal welfare was routinely checked by veterinarians from the Service for Biotechnology and Animal Welfare (Istituto Superiore di Sanita' (ISS) and Catholic University) and every effort was made to minimize the number of animals used and their suffering. Humane endpoint was set in keeping with the general well being or behavior of the mice. The experimental protocols were approved by the ISS and the Catholic University Ethical Committees.

### Mice

IRF-8-deficient (^−/−^) mice on a C57BL/6 background [19] and their wild-type counterparts (WT-B6) were bred and housed in a pathogen-free animal facility at ISS.

### Microorganisms

Pathogenic *Mtb* Erdman strain was grown in Middlebrook 7H9 broth supplemented with ADC and Tween 80. Mycobacteria were harvested, resuspended in sterile PBS (pH 7.2) and stored at −80°C until use. Before infection, strain aliquots were grown on 7H10 plates supplemented with OADC to titer bacteria after thawing.

### Infections and CFU assay

Infection studies were performed in a Biosafety level-3 facility. Mice were aerogenously infected with ∼100 CFU *Mtb*/animal and maintained until they became moribund and necessitated euthanization. At 8, 15 and 30 days, groups of 5 mice were sacrificed and bacteria in spleens and lungs enumerated by CFU assay on 7H10/OADC plates. The *Mtb* colonies were counted after 21 days of incubation at 37°C in sealed plastic bags.

### Histopathologic analysis

The lung left lobes were perfused and fixed with 10% paraformaldehyde in PBS and then embedded in paraffin for sectioning. The tissue sections were stained with hematoxylin and eosin (H&E) reagents or with Ziehl-Neelsen acid-fast stain and the lesion morphology and distribution were evaluated by light microscopy. For each lung left lobe, at least six sections were obtained and five mice per group were analyzed (total thirty lung sections per experimental group). For each section, the total surface area and the area with lesions were measured and averages calculated for each section and for each group (five lung left lobes per group). The slides were imaged using Nikon Eclipse 80i microscope and digital computer images were recorded with a Nikon DS-L2 camera control unit and the Nikon dedicated software 3422.1001.1798.080117. Cellular automatic count and histological measurements were carried out using the dedicated software Axiovision ver. 4.4 (Zeiss) by two independent researchers on two independent photo series.

### Immunohistochemistry and Immunofluorescence analysis

For these procedures the following mAb were used: purified anti-CD3 and anti-CD8 (Santa Cruz Biotechnology); purified anti-CD4, anti-FDC-M1 and biotin anti-CD45R/B220 (BD Pharmingen); biotin anti-F4/80 and anti-7/4 (Caltag); purified anti-Foxp3 (eBioscience); purified anti-DEC205 (Dendritics); purified anti-CXCL12/SDF1beta and anti-CCL19/Mip3beta (Abcam). Lung sections (3 μm thick) from formalin-fixed, paraffin-embedded tissue were mounted on positively charged Superfrost slides (Fisher Scientific). Tissue sections were deparaffinized and rehydrated through a series of graded alcohols. Antigens were retrieved by a high-temperature heating method. Briefly, slides were immersed in target retrieval solution at pH 6 (Dako), in a steamer (90–95°C) for a 20-minute incubation for all antigens. Tissues were then blocked for endogenous peroxidase in 3% hydrogen peroxide in water. Slides were then incubated in PBS containing 2% BSA, stabilizing protein and 0.015 mol/L sodium azide (Protein Block Serum-Free, Dako) to prevent non-specific binding. Tissues were incubated overnight at 4°C with primary mAbs, followed by secondary Ab for 1 h at r.t. For immunohistochemistry tissues were incubated with the streptavidin–biotin–peroxidase complex (Lab Kit peroxidase, Dako) at r.t. for 45 min and the reaction was revealed using the chromogen 3, 3′-diaminobenzidine (DAB) (DakoCytomation) and Fast Red (Sigma). Sections were counterstained with Mayer's hematoxylin and then cover-slipped in 50∶50 xylene/Permount (Fisher Scientific). For immunofluorescence, slides were incubated with Alexa Fluor® 555 streptavidin (Invitrogen) and then counterstained with blue Hoechst. Control slides, known to be positive for each antibody, were incorporated into each run. The slides were recorded with a Nikon DS-L2 camera control unit and the Nikon dedicated software 3422.1001.1798.080117 for immunohistochemistry analysis and Leica LAS AF (build 7266) for immunofluorescence analysis. Quantification of positive cells was performed by determining the area occupied by cellular infiltrates by means of the automated, morphometric tool of the Zeiss Axiovision ver. 4.4 (, (Zeiss), which determines the area defined by the squared micron value for each lung section measured as reported [25]. Morphometric analysis was performed in a blinded manner by two independent researchers on three independent photo series (at 400x magnification) of three independent experiments.

### Flow cytometry analysis

For phenotypical analyses, the following mAbs (from BD Pharmingen or eBioscience) were used: anti-CD45 PercP5.5; anti-CD11c-biotin; anti-SiglecF PE; anti-CD11b APC; anti-Gr1 APC-eFluor 780; anti-CD8 FITC; anti-CD3 APC; anti-CD4 FITC. Biotinylated mAbs were detected with streptavidin-eFluor 450 (eBioscience). Stained cells were analyzed on a FACSAria flow cytometer (Becton Dickinson). Cell populations (CD45^+^-gated) were determined as follows: granulocytes, CD11c^−^CD11b^+^Gr-1^+^; DC, CD11c^+^SiglecF^−^Gr-1^−^; macrophages, CD11c^+^SiglecF^+^ CD11b^low/−^; CD4^+^ T cells, CD3^+^CD4^+^; CD8^+^ T cells, CD3^+^CD8^+^.

### Statistical analysis

Significance for comparison between samples was determined by two-tailed Student's t-test after checking the normal distribution of data with Shapiro-Wilk test. Statistical analyses were performed using Stata 11.2 software (StataCorp, College Station, TX, USA) and P values less than 0.05 were considered statistically significant.

## Results

### Uncontrolled growth of pulmonary granulomas at late phase of *Mtb* infection in IRF-8^−/−^ mice

**Figure 1:**
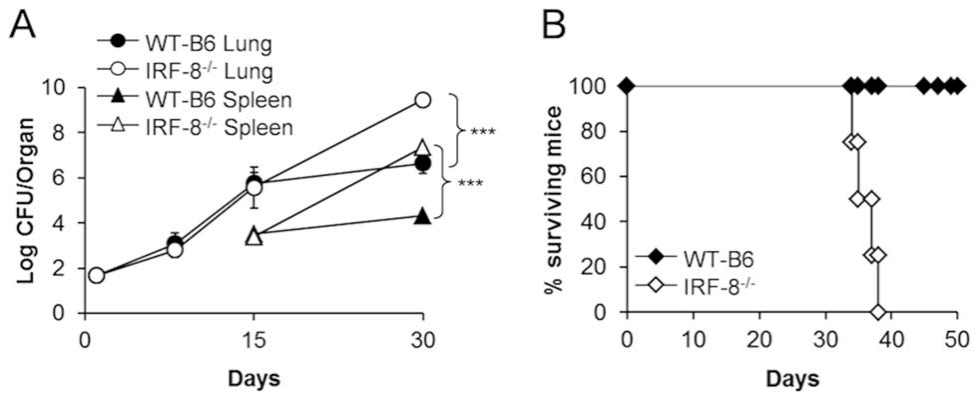
Increased susceptibility of IRF-8^−/−^ mice to *Mtb* infection. IRF-8^−/−^ and WT-B6 mice were infected with 100 CFU *Mt*b via the aerosol route. A) Bacterial loads, determined as CFU, in spleens and lungs at days 8, 15 and 30 p.i. Data are expressed as mean ± SEM of each individual mouse (n = 5 mice/group). ***P<0.001 IRF-8^−/−^ *vs.* WT-B6 at day 30 p.i. B) Surviving mice (n = 5 mice/group). One representative experiment out of three is shown.

**Figure 2:**
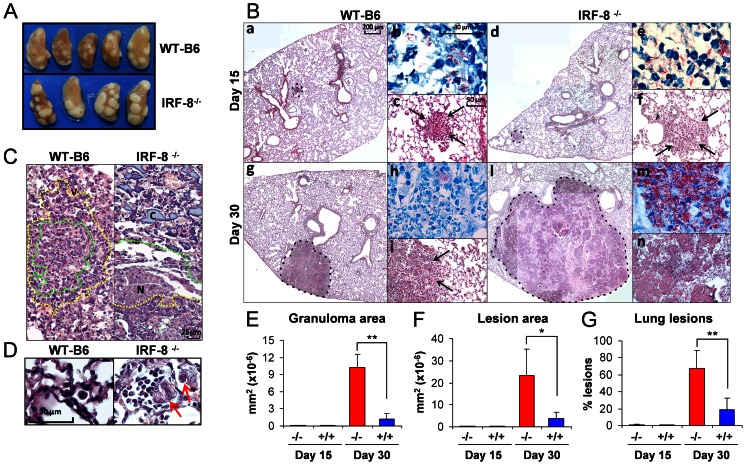
Uncontrolled growth of pulmonary granulomas at late phase of *Mtb* infection in IRF-8^−/−^ mice. A) Macroscopic appearance of pathology of lungs in IRF-8^−/−^ and WT-B6 mice at day 30 p.i. B) Histopathological analysis of lung sections colored with H&E at days 15 and 30 p.i.. Dotted lines in panels a, d, g, l (20X magnification) show granuloma formation. Panels c, f, i, n show a 200X magnification of the granuloma. Arrows depict granulomatous structures. Panels b, e, h, m (1000X magnification) show Ziehl-Neelsen acid-fast staining. C) Detail of granulomatous lesions at day 30 p.i. showing lymphocyte area (dotted yellow lines) and macrophage area (dotted green lines). V: blood vessel; N: coagulative necrosis; C: colliquative necrosis (400X magnification). D) H&E stained detail of macrophage area in granulomas (1000X) at day 30 p.i. showing foamy cells (red arrow). E–G) Extent of tissue damage assessed by E) median granuloma surface area; F) median total tissue surface area with lesions and G) percentage of tissue surface area with lesions with respect to total lung area. Five lungs per group were analyzed with at least three sections in two different points of the sample. Representative slides are shown. *P<0.05; **P<0.01.

IRF-8^−/−^ and immunocompetent WT-B6 mice were aerogenously infected with low-dose *Mtb* Erdman strain ( Fig. S1A) [26]. Up to day 15 post infection (p.i.) the two groups of mice exhibited comparable *Mtb* replication, with bacterial loads reaching similar levels in WT-B6 and IRF-8^−/−^ spleens and lungs (Fig. 1A). By day 30 p.i., while WT-B6 mice were able to contain bacterial growth, IRF-8^−/−^ mice exhibited exponential *Mtb* replication, reaching 9.4 logs CFU in lungs and 7.3 logs CFU in spleens accounting respectively for 2.8 log and 3 log-higher bacterial loads compared to controls (Fig. 1A). IRF-8^−/−^ mice eventually succumbed to *Mtb* infection (Fig. 1B), in contrast to WT-B6, which survived and developed a classical chronic/persistent infection [27]. Macroscopical analysis of lungs at day 30 p.i. showed the presence of larger tubercles in IRF-8^−/−^ mice compared to controls, indicative of massive cellular infiltration at the site of bacterial replication (Fig. 2A). H&E staining of lung sections at day 15 p.i. evidenced few granulomas in healthy lung parenchyma of both WT-B6 and IRF-8^−/−^ mice (Fig. 2B panels a, d), albeit those of deficient mice appeared less organized with a increased number of mononuclear cells infiltrated (Fig. 2B panels c, f). At day 30 p.i., giant granulomas with poor structural organization and extensive necrotic areas within widespread destructed lung parenchyma were observed in IRF-8^−/−^ but nor in WT-B6 mice (Fig. 2B panels g, l, i, n). In particular, granulomas of WT-B6 mice exhibited a compact structure with an inner layer composed by large epithelioid cells and intact phagocytes and an outer layer with abundant lymphocytes (Fig. 2C). In contrast, this structure was completely disrupted in IRF-8^−/−^ mice, with loss of inner and outer layers and appearance of extensive areas of colliquative and coagulative necrosis showing a more advanced stage of the disease (Fig. 2C). Moreover, multifocal foamy macrophages, many of which lysed, harboring high bacterial loads were found in granulomas of IRF-8^−/−^ mice (Fig. 2B panel m, 2D). Instead, well-organized non-necrotizing granulomatous lesions characterized by macrophages containing few intracellular bacilli were observed in WT-B6 lungs (Fig. 2B panel h, 2D). Accordingly, quantitative analysis of tissue damage revealed that, although the numbers of granulomas in the lungs did not significantly differ between mouse strains (data not shown), at day 30 p.i. IRF-8^−/−^ mice exhibited significantly higher median granuloma surface area (Fig. 2E), median total tissue surface area with lesions (Fig. 2F) and area with lesions versus healthy tissue ratio (Fig. 2G) compared to controls. Hence, IRF-8 loss-of-function is detrimental in restricting bacterial replication following aerogenous infection suggesting its causative role in lung pathogenesis occurring at late stages after *Mtb* entry.

### Defective recruitment of T lymphocytes and prevalence of neutrophils characterize acute TB in IRF-8^−/−^ mice

**Figure 3:**
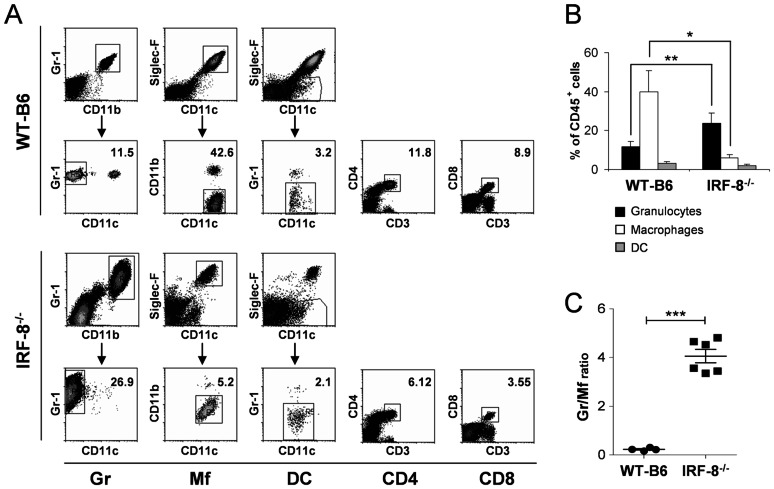
Immune cell distribution in lungs of uninfected IRF-8^−/−^ *vs.* WT-B6 mice. Lung cells from naïve IRF-8^−/−^ and WT-B6 mice were labelled with a panel of fluorescent mAbs against the indicated surface markers. A) Representative FACS analysis of indicated cell populations. Upper panels show cells gated over total CD45^+^ leukocytes. Plots in lower panels show cell populations gated as indicated by the arrows. Numbers represent the percent values of each indicated cell population over total CD45^+^cells. B) Mean percentages of lung granulocytes, macrophages and DC ±SD (n = 6 mice/group). *P<0.05; **P<0.01. C) Ratio values between granulocytes and macrophages ±SEM (n = 6 mice/group). ***P<0.001. Data are representative of one experiment out of four.

We investigated whether the greater bacterial burden in lungs of IRF-8^−/−^ compared to WT-B6 mice was associated with differential inflammatory infiltrates. First, we characterized the immune cell composition in naive IRF-8^−/−^ and WT-B6 lungs and found significantly increased frequencies of Gr-1^+^CD11b^+^CD11c^−^ granulocytes in deficient mice compared to controls (26.9% *vs.* 11.5% of total CD45^+^ leukocytes; Fig. 3A). Conversely, SiglecF^+^CD11b^low^CD11c^+^ macrophages were drastically reduced in IRF-8^−/−^ compared to WT-B6 animals (5.2% *vs.* 42.6% of total CD45^+^ cells). Moreover, IRF-8^−/−^ lungs displayed reduced SiglecF^−^Gr1^−^CD11c^+^ DC, CD3^+^CD4^+^ and CD3^+^CD8^+^ T lymphocytes with respect to controls (Fig. 3A). Quantitative analysis revealed granulocytes representing the predominant innate immune population in IRF-8^−/−^ lungs, as opposed to WT-B6 organs, where macrophages prevailed (Fig. 3B), resulting in a granulocyte/macrophage ratio 4-fold higher in deficient mice compared to controls (Fig. 3C).

**Figure 4:**
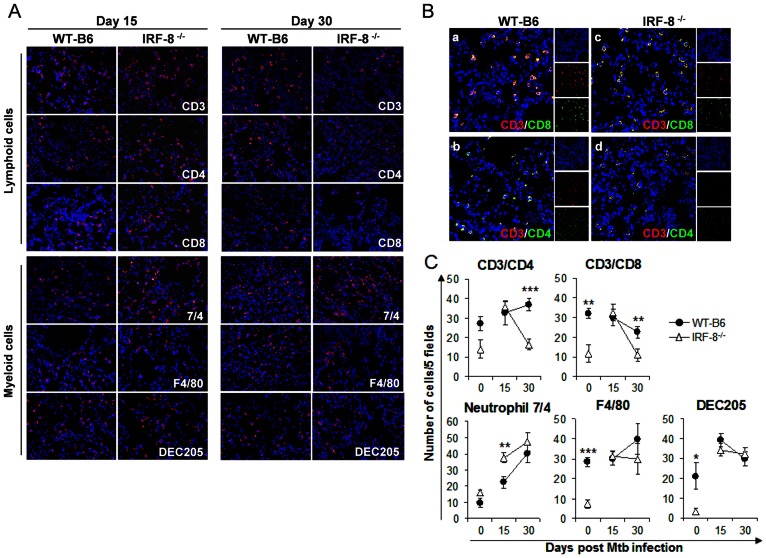
Altered immune infiltration in lungs of *Mtb*-infected IRF-8^−/−^ mice. A) Representative CLSM analysis of lung tissues from IRF-8^−/−^ and WT-B6 mice at days 15 and 30 p.i. stained with a panel of mAbs against the indicated markers B) Representative CLSM analysis of lung sections labeled with anti-CD3 plus either anti-CD4 or anti-CD8 Abs. C) Morphometric analysis cells expressing the indicated markers in each individual lung section. Data express mean cell numbers ±SD of 5 fields (1 field was 0.16 mm^2^ at 400x magnification) of each slide (4–15 slides/mice; n = 3 mice/group) analyzed in lungs of *Mtb-*infected IRF-8^−/−^ and WT-B6 and their uninfected counterparts. One representative experiment out of two is shown. *P<0.05; **P<0.01; ***P<0.001.

Next, we assessed immune cell trafficking in lungs after *Mtb* infection. Immunofluorescence analysis revealed that despite the reduced frequencies of DC, macrophages and CD4^+^ and CD8^+^ T cells in uninfected IRF-8^−/−^ mice with respect to WT-B6 counterpart, following Mtb infection at day 15 p.i. these immune populations were efficiently recruited in the lungs of deficient mice at the same extent of WT-B6 animals, suggesting the existence of compensatory mechanisms in the former group (Fig. 4A–C). Nevertheless, at day 30 p.i. the frequencies of CD4^+^ and CD8^+^ T cells decreased sharply in lungs of IRF-8^−/−^ mice whereas DEC205^+^ DC and F4/80^+^ macrophages remained in similar frequencies in the two mouse strains (Fig. 4A–C). In contrast, 7/4^+^ neutrophils, already increased in uninfected deficient lungs with respect to immunocompetent counterpart, accumulated at higher levels in IRF-8^−/−^ lungs with respect to WT-B6 organs by day 15 p.i. (Fig. 4A, C). Of interest, IRF-8^−/−^ lungs also exhibited reduced infiltration of Foxp3^+^ Treg at day 15 p.i. and, more evidently, at day 30 p.i. compared to WT-B6 controls (Fig. S2A, B). These results indicate that uncontrolled growth of *Mtb* in lungs of IRF-8^−/−^ mice is associated with a loss of pulmonary recruitment of CD3^+^CD4^+^, CD3^+^CD8^+^ T lymphocytes at late stages of infection. On the other hand, the high levels of neutrophils found in lungs of deficient mice support a role for IRF-8 in controlling the inflammatory ability of these cells in TB pathogenesis.

### Impaired development of LS in IRF-8^−/−^ lungs after *Mtb* infection

**Figure 5:**
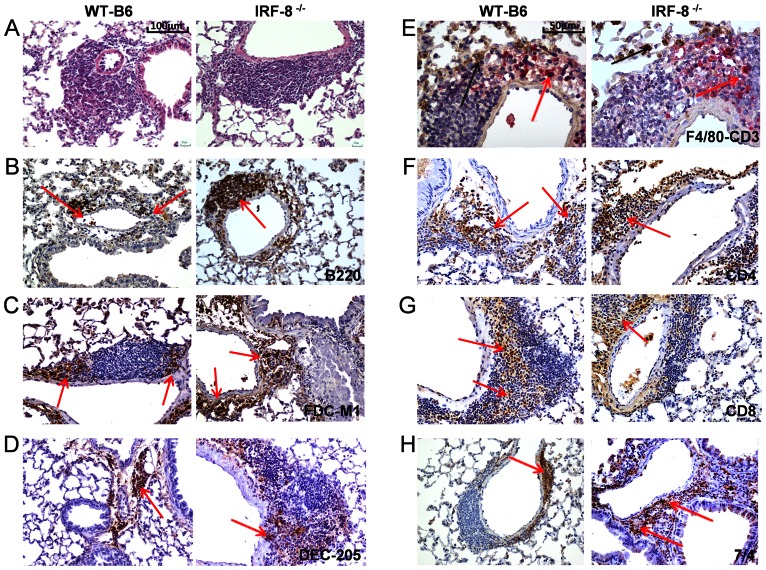
Formation of LS in the lungs of *Mtb*-infected IRF-8^−/−^ *vs.* WT-B6 mice. IHC analysis of pulmonary sections from *Mtb*-infected mice at day 15 p.i. showing the distribution of immune cells in perivascular, peribronchial and interstitial areas of lung tissues. A) H&E staining (200X). B–D and F–H) Staining with the indicated mAbs (brown). Red arrows depict marker-positive cells. E) Co-staining with anti-F4/80 (brown) and anti-CD3 (red) mAbs, depicted by black and red arrows respectively. Representative slides are shown, all taken at 200X magnification, except for (E) taken at 400X magnification. Three independent experiments were performed.

**Figure 6:**
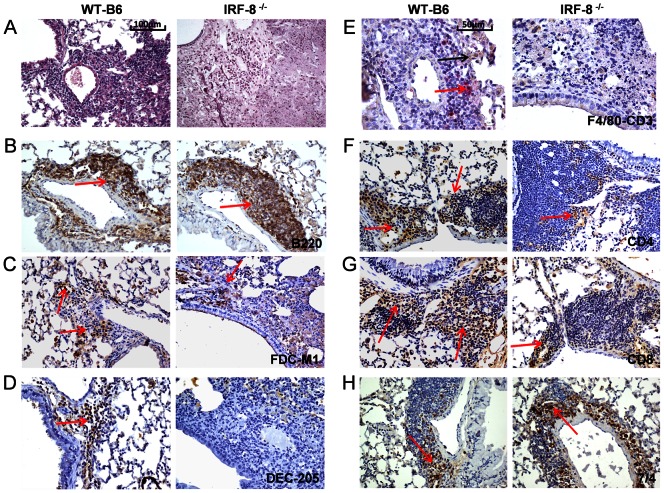
Loss of LS organization in lungs of *Mtb*-infected IRF-8^−/−^. IHC analysis of pulmonary sections from *Mtb*-infected mice at day 30 p.i. showing the distribution of immune cells in perivascular, peribronchial and interstitial areas of lung tissues. A) H&E staining (200X). B–D and F–H) Staining with the indicated mAbs (brown). Red arrows depict marker-positive cells. E) Co-staining with anti-F4/80 (brown) and anti-CD3 (red) mAbs, depicted by black and red arrows respectively. Representative slides are shown all taken at 200X magnification, except for (B) taken at 400X magnification. Three independent experiments were performed.

LS neogenesis in lungs has been reported to influence the development of acquired immunity after *Mtb* infection [5]. Therefore, we evaluated whether the peculiar inflammatory infiltration in the lung parenchyma of *Mtb*-infected IRF-8^−/−^ correlated with the defective occurrence and/or structural organization of LS. At day 15 p.i., accumulation of immune infiltrates in perivascular, peribronchial and interstitial areas indicative of LS were observed in both mouse strains (Fig. 5A). In particular, in LS of IRF8^−/−^ mice B220^+^ cells were identified in bigger aggregates, suggesting the presence of enlarged B cell areas, compared to the WT-B6 counterpart (Fig. 5B). Stromal follicular dendritic cells (FDC), essential for the generation of germinal centers (GC) in secondary lymphoid tissues [28], were found in well-organized aggregates and in defined lymphocytic enriched areas surrounding perivascular and peribronchial area only in WT-B6 mice (Fig. 5C). Conversely, FDC were interspersed within other immune infiltrates in lungs of IRF-8^−/−^ mice, further indicating, a less organized architecture of LS (Fig. 5C). To better analyze the cellular organization of LS we performed double staining for F4/80^+^ macrophages and CD3^+^ T lymphocytes (Fig. 5E). We found a comparable infiltration of CD3^+^T lymphocytes, both CD4^+^ and CD8^+^ T cells, as well as similar levels of F4/80^+^ macrophages in the areas surrounding vessels and bronchial tree in both IRF-8^−/−^ and WT-B6 lungs (Fig. 5E, F, G). In addition, DEC205^+^ DC accumulated at similar frequencies in areas surrounding pulmonary vessels and bronchi of both strains of mice (Fig. 5D). In contrast, higher numbers of 7/4^+^ neutrophils aggregates were found in LS of IRF-8^−/−^ mice compared to controls (Fig. 5H). Of interest, while the areas surrounding the bronchial tree of WT-B16 lungs showed consistent recruitment of Foxp3^+^ Treg cells, this population was barely detectable in IRF-8^−/−^ tissues (Fig. S2C). At day 30 p.i., the lung architecture in IRF-8^−/−^ mice was massively altered, LS appeared disorganized and immune infiltrates dispersed within damaged parenchyma, whereas in WT-B6 LS appeared well conserved (Fig. 6A). Despite the diffuse destruction of pulmonary tissue, B220^+^ cells were still localized as bigger aggregates in perivascular and peribronchial areas of IRF-8^−/−^ lungs as compared to WT-B6 (Fig. 6B). In contrast, CD3^+^ T lymphocytes, both CD4^+^ and CD8^+^ T cells, declined massively in LS of IRF-8^−/−^ mice with respect to WT-B6 structures, as evidenced by the scattered signal across the lung parenchyma (Fig. 6E–G). At the same extent, while F4/80^+^ macrophages, FDC and DC were found in well-defined aggregates within WT-B6 LS, their signals were barely detectable within IRF-8^−/−^ ones (Fig. 6C–E). Notably, 7/4^+^ neutrophils were retained at elevated frequencies in perivascular and peribronchial areas of IRF-8^−/−^ lungs (Fig. 6H).

**Figure 7:**
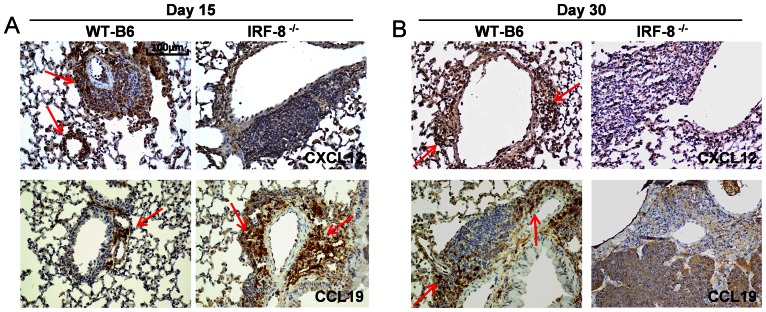
LS organization in lungs of *Mtb*-infected mice correlates with in situ chemokine expression. IHC analysis of peribronchial and interstitial areas of lung tissues from *Mtb*-infected IRF-8^−/−^ and WT mice at day 15 (A) and day 30 p.i. (B) after staining with the indicated mAbs (brown) against CCL19 and CXCL12. Red arrows depict marker-positive cells. Representative slides are shown all taken at 200X magnification. Three independent experiments were performed.

Because the homeostatic chemokines CCL19 and CXCL12 are required for the organization of lymphoid areas in the lungs [29], we next examined their expression in pulmonary LS of IRF-8^−/−^ and WT-B6 mice following *Mtb* infection. As shown in Fig. 7A, at day 15 p.i. pulmonary tissue of IRF-8^−/−^ mice exhibited higher staining for CCL19 but lower CXCL12 expression as compared with the WT-B6 counterpart. However, at day 30 p.i. neither CCL19 nor CXCL12 could be detected in lungs of IRF-8^−/−^ mice, while both chemokines were highly expressed in WT-B6 animals (Fig. 7B). These findings suggest a close correlation between pulmonary CCL19 and CXCL12 expression and the organization of LS throughout Mtb infection.

### Defective maintenance of immune cell recruitment in lung granulomas of IRF-8^−/−^ mice

**Figure 8:**
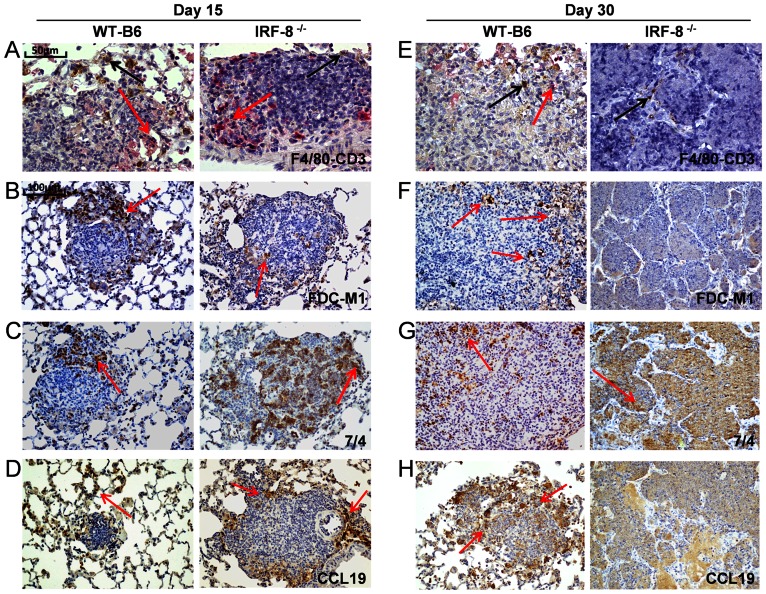
Immune infiltrates into granulomas of *Mtb*-infected IRF-8^−/−^ *vs.* WT-B6 mice. IHC analysis of pulmonary sections from *Mtb*-infected mice at days 15 and 30 p.i. showing the distribution of immune cells within granulomatous lesions. A, E) Co-staining with anti-F4/80 (brown) and anti-CD3 (red) mAbs, depicted by black and red arrows respectively. B–D, F–H) Staining with the indicated mAbs (brown). Red arrows depict marker-positive cells. Representative slides are shown all taken at 200X magnification, except for A and E taken at 400X magnification. Three independent experiments were performed.

The infiltrates in granulomatous lesions of IRF-8^−/−^ and WT-B6 mice were analyzed. At day 15 p.i., CD3^+^ T lymphocytes were still detectable in the outer layers of IRF-8^−/−^ granulomatous structures (Fig. 8A). In contrast, while in WT-B6 granulomas numerous FDC were detected in a well-localized reticulum, few FDC were identified interspersed within IRF-8^−/−^ granulomas (Fig. 8B). Remarkably, 7/4^+^ neutrophils accumulated in a diffuse pattern in much higher frequency in granulomas of IRF-8^−/−^ group with respect to WT-B6 controls (Fig. 8C). Of interest, at this time point IRF-8^−/−^ granulomas exhibited high levels of CCL19 in the outer layers (Fig. 8D). At day 30 p.i., while WT-B6 granulomas maintained structural integrity with detectable levels of CD3^+^ T lymphocytes, F4/80^+^ macrophages and FDC, IRF-8^−/−^ granulomatous lesions displayed a disorganized caseous structure, with extensive necrotic areas and few infiltrating T cells, macrophages and FDC aggregates (Fig. 8E, F). Nonetheless, elevated amounts of 7/4^+^ neutrophils persistently populated IRF-8^−/−^ granulomas (Fig. 8G). In parallel, in situ CCL19 expression markedly declined in IRF-8^−/−^ granulomatous lesions, whereas it increased in WT-B6 structures (Fig. 8H). Finally, although high accumulation of B220^+^ B lymphocytes in IRF-8^−/−^ granulomas was observed at 15 day p.i. these cells were not detected at day 30 p.i. (Fig. S3). Together, these results suggest that although early recruitment of T cells and of myeloid cell populations, such as macrophage, FDC and DC was not impaired *per se* in granulomas of IRF-8^−/−^ mice, their frequency within these structures gradually declined during disease progression. In parallel, the distinctive disorganized growth of IRF-8^−/−^ granulomas was associated to a massive recruitment and persistence of neutrophils throughout TB disease.

## Discussion

Recently, a genetic approach revealed that heritable mutations in IRF-8 gene determine human primary immunodeficiency, which affects the functional activity of DC, resulting detrimental in anti-mycobacterial immune response [24]. Indeed, the importance of IRF-8 in immunity against mycobacteria was already revealed by previous studies identifying this factor as a critical regulator of host defenses against BCG and, in some settings, against *Mtb* [30]. BXH-2 mice, bearing a defective IRF-8R394C allele, develop disseminated and rapidly fatal TB following systemic *Mtb* infection [23]. In this study we have extended these observations by dissecting the immune components occurring in lungs of IRF-8^−/−^ mice following *Mtb* aerogenic infection. We report three crucial events characterizing *Mtb* infection in IRF-8^−/−^ mice that result in fatal acute TB: i) an uncontrolled growth and disorganized structure of granulomas distinguished by altered immune infiltration and progressive loss of T lymphocytes leading to the inability to immunologically restrain bacterial replication at late stages; ii) an impaired development of pulmonary LS associated with the failure to maintain a proper T cell recruitment within these structures, underlying the importance of these components for TB progression; iii) a persistent homing of neutrophils into both LS and fast-growing granulomas, suggesting their crucial contribution to the development of acute TB.

It is generally accepted that immune responses at the infection site reflect immunity in the periphery concurring to TB control [31]. About 2–3 weeks p.i. as T lymphocytes are recruited in granulomas, adaptive immunity is generated as a result of multiple factors, including type and abundance of DC, which determine the strength and persistence of immune response [32]. If these events fail to occur, *Mtb*-specific immune response is not established, causing disease progression [7]. Protective immune responses can be generated independently of secondary lymphoid organs during *Mtb* infection and implicate local pulmonary T-cell priming as a crucial mechanism for host defense onset [33]. In this regard, in both humans and mice, *Mtb* infection has been associated with the neogenesis of pulmonary LS [2], [34]. Some studies reported that granulomas ensuing from *Mtb* infection also contain lymphoid areas resembling a form of neo-generated LS [9], [35]. Our data show that truthful organization of granulomas and pulmonary LS are processes tightly controlled by IRF-8, since they occur properly in lungs of *Mtb-*infected immunocompetent but not in IRF-8-deficient mice. As a consequence, IRF-8^−/−^ mice were found to be extremely susceptible to aerosol infection with low-dose *Mtb* Erdman strain, succumbing to fatal acute TB within day 40 p.i., whereas the immunocompetent animals contained the infection switching from acute to chronic TB. We demonstrate that the breakpoint at which the course of TB diverges in the two strains of mice is 15 days p.i., when the onset of adaptive immunity is expected to occur [36], and this event is unrelated to the early pulmonary recruitment of key innate immune populations. In fact, up to day 15 p.i., bacterial growth and dissemination in lungs and spleens were comparable in both animals. However, by this time point a different balance of immune cell subsets already existed between lungs of IRF-8^−/−^ and WT-B6 mice. In particular, with respect to WT-B6 mice, IRF-8^−/−^ animals exhibited a more marked neutrophil accumulation in lungs predictive of acute inflammation [37]. However, IRF-8^−/−^ mice showed percentages of pulmonary macrophages, DC, CD4^+^ and CD8^+^ T cells substantially similar to those of WT-B6 mice, suggesting an accelerated and compensatory pulmonary recruitment of these populations at early stage of infection in deficient animals. The concomitant decrease of Treg cells suggests the prevalence of traits predisposing to exuberant inflammatory response more than immune protection in IRF-8^−/−^ lungs [6]. Importantly, the unbalanced presence of certain immune cells in the lungs of IRF-8^−/−^ mice differently reflects their distribution within LS and granulomas throughout TB. At day 15 p.i. efficient recruitment of multiple populations, including macrophages, DC, FDC, B cells, CD4^+^ and CD8^+^ T lymphocytes occurred within LS of IRF-8^−/−^ mice. However, these LS were poorly organized, failed to display separated T and B cell zones resulting in loss of immune infiltrates and LS disruption at late stage of disease. On the contrary, early after *Mtb* infection WT-B6 LS exhibited well-defined B cell areas associated to FDC aggregates along with efficient recruitment of immune cells [29] and maintained a defined organization throughout TB. Importantly, the high expression of CCL19 in IRF-8^−/−^ LS at day 15 p.i. correlated with the accumulation of immune infiltrates but not with the organization of these structures. At the same time point, high levels of CXCL12 in WT-B6 LS correlate with the recruitment and spatial localization of the immune infiltrates. At late phase of infection, high expression of both CCL19 and CXCL12 was observed only in control mice and correlated with the maintenance and the lymphoid organization of immune infiltrates composed of CD4^+^ and CD8^+^ T cells, macrophages, DC and FDC. These findings suggest that homeostatic chemokines such as CCL19 and CXCL12 have non-overlapping roles in their ability to promote LS organogenesis, confirming previous reports [38], [39]. Thus, at early stage of infection high levels of CCL19 in IRF-8^−/−^ mice may account for the pulmonary recruitment of immune infiltrates, possible through a chemokine/lymphotoxin loop [40]. In contrast, the spatial organization of well-defined T and B cell zones in LS depends on additional signals including high levels of CXCL12 that are induced in WT-B6 controls, but not in IRF-8^−/−^ mice.

IRF-8 deficiency does not appear to limit the initial steps of granuloma formation, as evidenced by efficient recruitment of immune cells including macrophages, T lymphocytes and FDC in deficient mice at early stages. The occurrence of these events is in accordance with the high levels of CCL19 an may lead to the fast development of granulomatous structures, which are likely exploited by mycobacteria for their proliferation and dissemination within the host [41]. However, at late stages of infection, when IRF-8^−/−^ granulomas become acute exhibiting features of caseous necrosis, the influx of selected immune cells declined, causing failure of granuloma contain mycobacterial dissemination.

One important finding reported in this study is the massive infiltration of neutrophils in both LS and granulomas of IRF-8^−/−^ mice and their persistence at all stages of TB. Recently, IFN-inducible neutrophil activity in host response to *Mtb* has gained renewed interest [42]. Although early recruitment of neutrophils to sites of infection should ensure protective immunity, inefficiency in their activity may lead to detrimental immune responses [43]. In this light, high numbers of neutrophils early after *Mtb* infection are thought to correlate with protection, whereas at late stages of TB they may be associated to poor prognosis. Herein, we report that, despite the high numbers of lung neutrophils in the early stages of infection, a protective immunity was not established in IRF-8^−/−^ mice. We envisage that although these cells may restrict mycobacterial replication as suggested by similar bacterial loads in both strains of mice up to day 15 p.i., they may dampen the development of *Mtb*-specific immunity. In this regard, activated neutrophils have been reported to exert an immunosuppressive function on T cells in humans [44]. On the other hand, neutrophils can release cytokines, such as IL-12, and chemokines, such as CXCL10, that attract and activate T lymphocytes [45], or may deliver activation signals to DC, thus promoting protective immunity [1]. Since IRF-8 deficiency is associated to impaired IL-12 production and DC maturation [14], [21], the neutrophils recruited in *Mtb*-infected IRF-8^−/−^ lungs, may be unable to exert their function and to elicit protective adaptive immunity. The finding that also Treg cells were reduced throughout the course of infection in immunodeficient animals is consistent with the idea that, besides inflammation, IL-2-producing effector T cells drive the expansion of *Mtb*-specific Treg that limit tissue inflammation and damage [46], and suggest that IRF-8 controls several components crucial for the induction of adaptive immune response. Hence, at late stages of *Mtb* infection the clear-cut loss of CD4^+^ and CD8^+^ T cell infiltrates and the disruption of LS and granuloma structures of IRF-8^−/−^ lungs may represent key events hampering the initiation and amplification of a protective adaptive immunity.

Collectively, our results show that IRF-8 deficiency defines an acute mouse model of experimental TB. The pathological and immunological dissections of this model demonstrate that the proper formation of pulmonary newly-formed LS and granulomas is necessary to contain *Mtb* infection and limit TB disease. Moreover, our data disclose the prominent role of IRF-8 at late stages of *Mtb* infection, when the massive recruitment of neutrophils and the loss of T cells in both LS and granulomas define the poor outcome of TB in deficient mice. Lastly, given the recent identification of an IRF-8-dependent genetic control for human mycobacterial disease [24], this work highlights the importance of the IRF-8^−/−^ mouse model of acute TB as a valuable *in vivo* tool for comparative studies with chronically-infected congenic WT-B6 for dissecting those mechanisms underlying different immune reactions observed also in patients.

## Supporting Information

Figure S1 **Schedule of** ***Mtb*** **infection and sampling.** (A) IRF-8^−/−^ and WT-B6 mice were aerogenously infected with *Mtb* Erdman strain (∼100 CFU *Mtb*/animal). Determination of bacterial burden and histopathology of lungs were performed at 1, 2 and 4 weeks p.i. (B) Course of *Mtb* infection in WT-B6 mice. Time course of bacterial burden in lung and spleen at 7, 14, 28 and 70 days following aerogenous *Mtb* Erdman infection. Results are expressed as mean CFU ± SD of 5 mice per time point.
(TIF)

Figure S2 **Detection of Treg cells in lungs of** ***Mtb*** **-infected IRF-8^−/−^** ***vs.*** **WT-B6 mice.** A) Formalin-fixed paraffin-embedded lung tissues from IRF-8^−/−^ and WT-B6 mice at days 15 and 30 p.i. were stained with mAbs against Foxp3 and analysed by CLSM. One representative experiment out of two is shown. B) Morphometric analysis of Foxp3-expressing cells in each individual lung section. Data represents mean cell numbers ±SD of five fields (1 field was 0.16 mm^2^ at 400x magnification) of each slide (4–15 slides/mice; n = 3 mice/group) analyzed in lungs of *Mtb*-infected IRF-8^−/−^ and WT-B6 and their uninfected counterparts. *P<0.05; ***P<0.001. C) IHC analysis of Foxp3 staining in peribronchial and interstitial areas of lung tissues from *Mtb*-infected IRF-8^−/−^ and WT-B6 mice at days 15 and 30 p.i. Representative slides, taken at 200X magnification, are shown. Three independent experiments were performed.
(TIF)

Figure S3 **B lymphocytes in pulmonary granulomas of** ***Mtb*** **-infected IRF-8^−/−^** ***vs.*** **WT-B6 mice.** Infected mice were euthanized respectively at 15 and 30 days p.i., when the pulmonary tissues were processed and sections were subjected to IHC with Abs for the lymphocytic marker B220. Three independent experiments were performed. Representative slides are shown, all taken at 200 X magnifications.
(TIF)
