# Correction: Interferon Regulatory Factor 8-Deficiency Determines Massive Neutrophil Recruitment but T Cell Defect in Fast Growing Granulomas during Tuberculosis

**DOI:** 10.1371/annotation/cbf9ae84-2f74-4d4d-b2e7-b39e7ebaaaa8

**Published:** 2013-09-19

**Authors:** Stefano Rocca, Giovanna Schiavoni, Michela Sali, Antonio Giovanni Anfossi, Laura Abalsamo, Ivana Palucci, Fabrizio Mattei, Massimo Sanchez, Anna Giagu, Elisabetta Antuofermo, Giovanni Fadda, Filippo Belardelli, Giovanni Delogu, Lucia Gabriele

A funding organization for the 12th and 14th authors was incorrectly omitted from the Funding Statement. The Funding Statement should read: "This work was funded by the European Community Seventh Framework Programme (Project acronym: HOMITB, Title: *Host and microbial molecular dissection of pathogenesis and immunity in tuberculosis*; Grant agreement no.: 200732) to LG; by Italian Association for Research against Cancer (AIRC) grants to LG and FB. The funders had no role in study design, data collection and analysis, decision to publish, or preparation of the manuscript." 

