# Supplementary figures and images for: Interferon Regulatory Factor 8-Deficiency Determines Massive Neutrophil Recruitment but T Cell Defect in Fast Growing Granulomas during Tuberculosis
Source: PLoS One. 2013 May 24;8(5):e62751. doi: 10.1371/journal.pone.0062751 (PMC3663794; doi:10.1371/journal.pone.0062751)

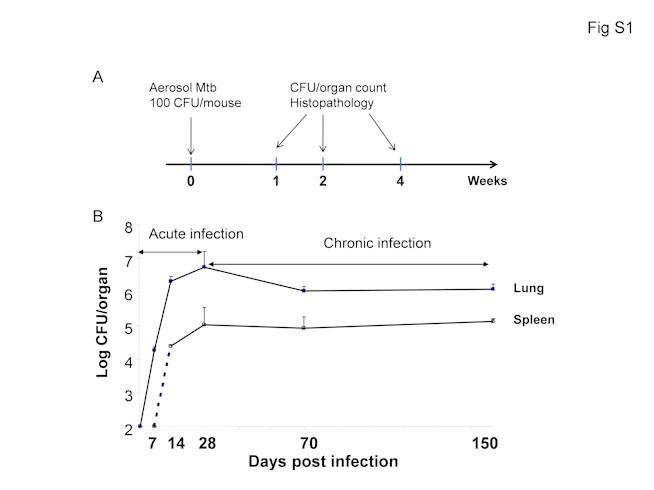

Supplement: Figure S1 — Schedule of Mtb infection and sampling. (A) IRF-8−/− and WT-B6 mice were aerogenously infected with Mtb Erdman strain (∼100 CFU Mtb/animal). Determination of bacterial burden and histopathology of lungs were performed at 1, 2 and 4 weeks p.i. (B) Course of Mtb infection in WT-B6 mice. Time course of bacterial burden in lung and spleen at 7, 14, 28 and 70 days following aerogenous Mtb Erdman infection. Results are expressed as mean CFU ± SD of 5 mice per time point. (TIF) [file pone.0062751.s001.tif]

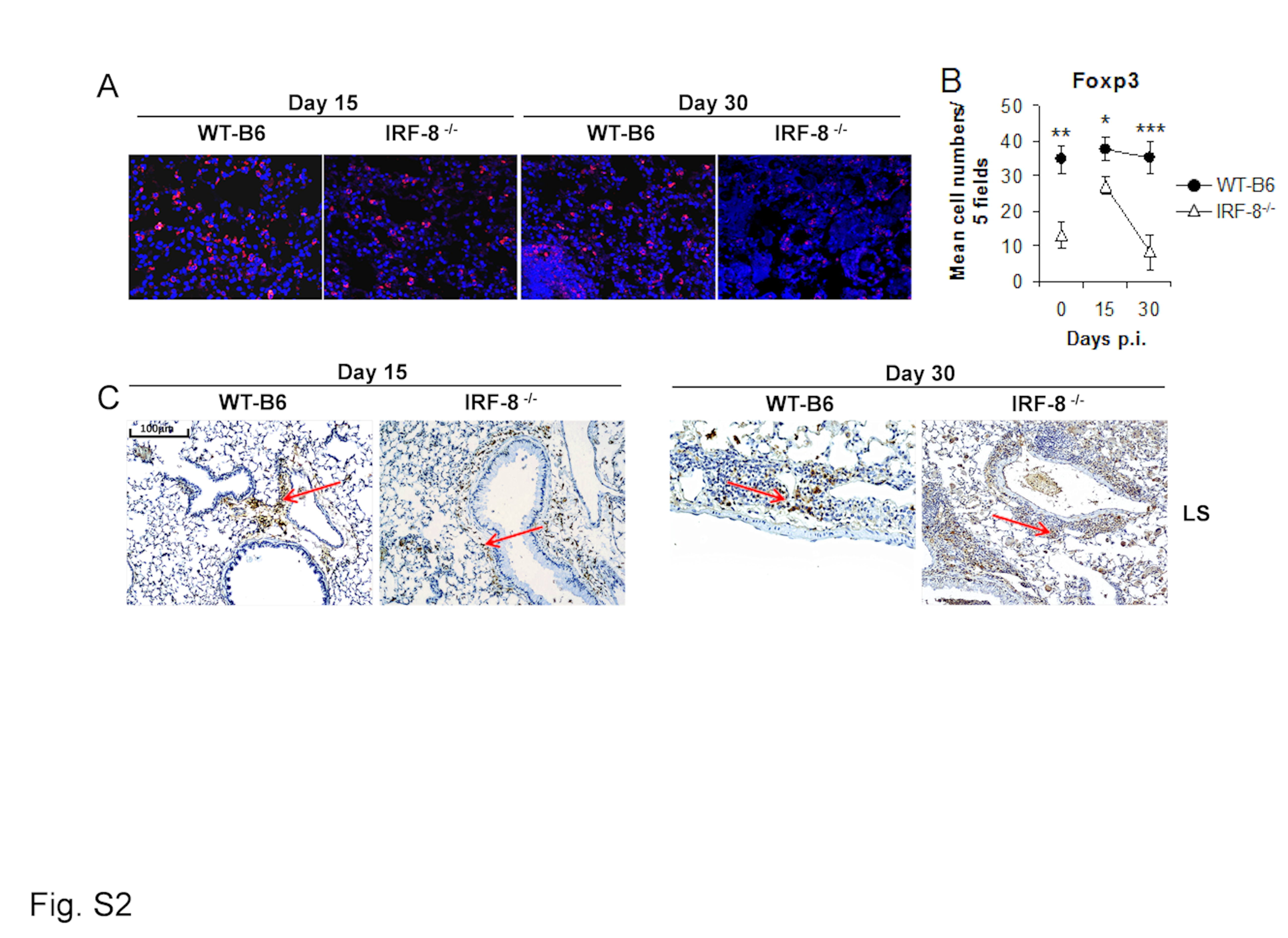

Supplement: Figure S2 — Detection of Treg cells in lungs of Mtb -infected IRF-8−/− vs. WT-B6 mice. A) Formalin-fixed paraffin-embedded lung tissues from IRF-8−/− and WT-B6 mice at days 15 and 30 p.i. were stained with mAbs against Foxp3 and analysed by CLSM. One representative experiment out of two is shown. B) Morphometric analysis of Foxp3-expressing cells in each individual lung section. Data represents mean cell numbers ±SD of five fields (1 field was 0.16 mm2 at 400x magnification) of each slide (4–15 slides/mice; n = 3 mice/group) analyzed in lungs of Mtb-infected IRF-8−/− and WT-B6 and their uninfected counterparts. *P<0.05; ***P<0.001. C) IHC analysis of Foxp3 staining in peribronchial and interstitial areas of lung tissues from Mtb-infected IRF-8−/− and WT-B6 mice at days 15 and 30 p.i. Representative slides, taken at 200X magnification, are shown. Three independent experiments were performed. (TIF) [file pone.0062751.s002.tif]

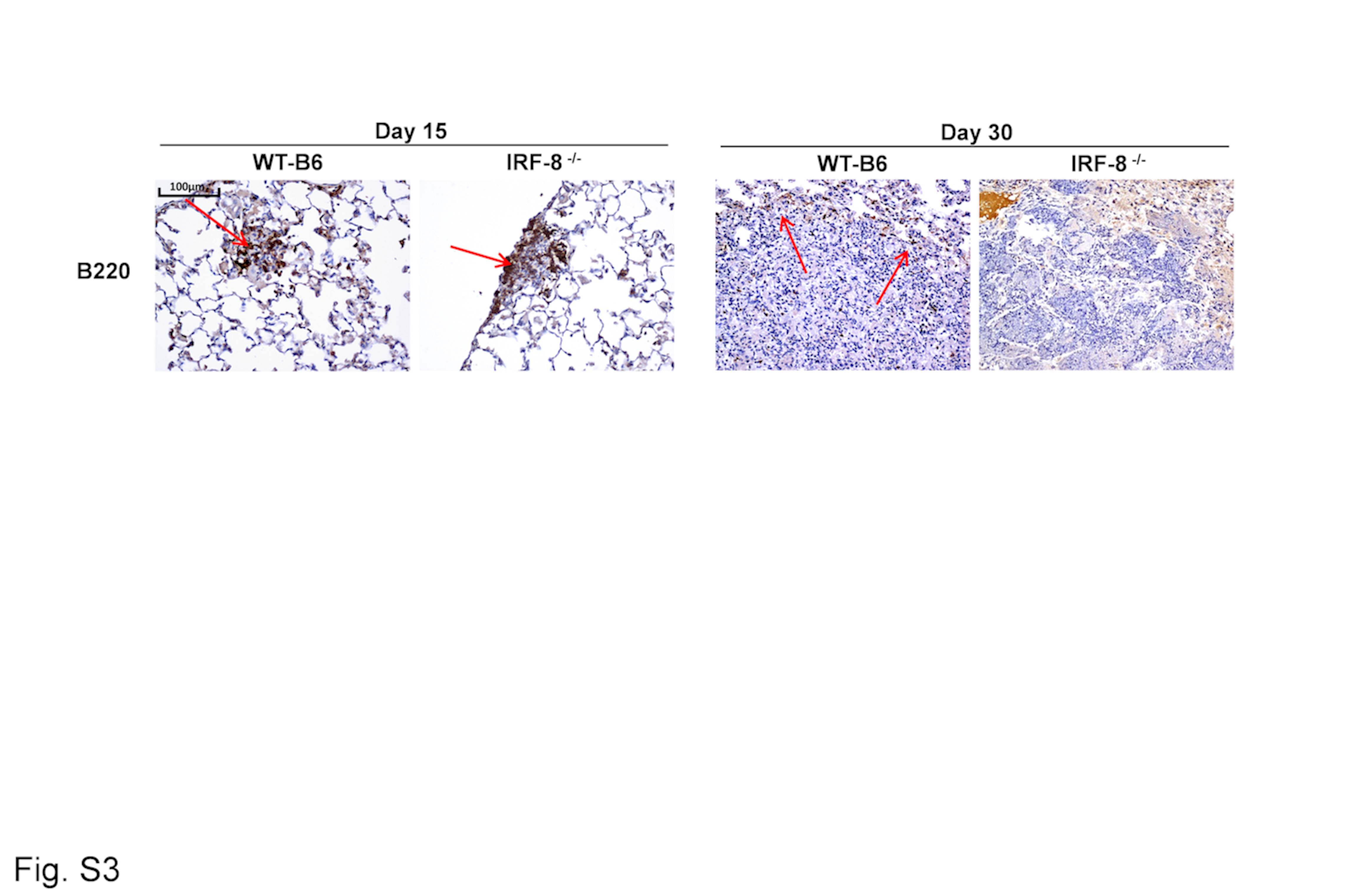

Supplement: Figure S3 — B lymphocytes in pulmonary granulomas of Mtb -infected IRF-8−/− vs. WT-B6 mice. Infected mice were euthanized respectively at 15 and 30 days p.i., when the pulmonary tissues were processed and sections were subjected to IHC with Abs for the lymphocytic marker B220. Three independent experiments were performed. Representative slides are shown, all taken at 200 X magnifications. (TIF) [file pone.0062751.s003.tif]
